# Longer Baseline Left Ventricular Activation Time Is Associated With Lower Mortality and Lower Risk of Heart Failure Hospitalization in Cardiac Resynchronization Therapy Recipients

**DOI:** 10.1111/anec.70210

**Published:** 2026-06-10

**Authors:** Sofia Marinko, Pyotr G. Platonov, Rasmus Borgquist

**Affiliations:** ^1^ Department of Cardiology, Clinical Sciences Lund University Lund Sweden; ^2^ Arrhythmia Clinic Skåne University Hospital Lund Sweden

**Keywords:** cardiac resynchronization therapy, heart failure, patient selection, prognosis

## Abstract

**Introduction:**

Many patients do not benefit from cardiac resynchronization therapy (CRT) with current guideline parameters. The objective of this study was to examine the relationship between left ventricular activation time (LVAT) from the standard 12‐lead surface electrocardiogram (ECG) and clinical outcome from CRT.

**Methods:**

A retrospective study was performed on patients receiving CRT implants at a large‐volume tertiary care center. Digital ECGs were collected pre‐ and post‐implant. LVAT was defined as the time from QRS onset to maximum deflection in lead V6. The primary combined endpoint was heart failure hospitalization or all‐cause mortality.

**Results:**

The study group comprised 415 patients (median age [Q1–Q3] of 72.8 years [65.1–78.7], 77.3% male, median baseline LVEF 27.5% [22–30], and 43.1% with ischemic heart failure etiology) who were followed for up to 7.6 years (median 2.8). LVAT was measured pre‐implant (median 78 ms [66–98]) and post‐implant (median 88 ms [74–106]). In Kaplan–Meier analysis, a longer pre‐implant LVAT was associated with a reduced risk of reaching the primary endpoint in patients with LBBB (log‐rank *p* = 0.046). Post‐implant LVAT was not associated with clinical outcome.

**Conclusion:**

Our results show that a longer baseline LVAT is associated with a lower risk of heart failure hospitalization and all‐cause mortality. This relationship was of borderline significance in multivariable analysis. Prospective trials would be useful to further explore the potential role of pre‐implant LVAT in patient selection for CRT.

## Graphical abstract

In patients undergoing CRT implantation, a longer pre‐implant LVAT was linked to better clinical outcome (lower risk of heart failure hospitalization and all‐cause mortality), suggesting that LVAT may help refine risk stratification after CRT.

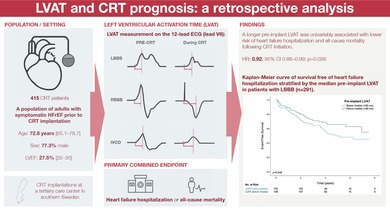

**Abbreviations**
CABG: coronary artery bypass grafting
CRT: cardiac resynchronization therapy
CRT‐D: cardiac resynchronization therapy with an additional function of an ICD
CRT‐P: cardiac resynchronization therapy pacemaker
ECG: electrocardiogram
eGFR: estimated glomerular filtration rate
ICD: implantable cardioverter‐defibrillator
IVCD: intraventricular conduction delay
LBBB: left bundle branch block
LV: left ventricle
LVAD: left ventricular assist device
LVAT: left ventricular activation time
LVEF: left ventricular ejection fraction
LVESV: left ventricular end‐systolic volume
NT‐proBNP: N‐terminal Pro–B‐type natriuretic peptide
NYHA: New York Heart Association
PCI: percutaneous coronary intervention
RBBB: right bundle branch block

## 1 Introduction

Cardiac resynchronization therapy (CRT) is an effective device for patients with systolic heart failure and dyssynchronous left ventricular (LV) activation (Brignole et al. 2013; Abraham et al. 2002). CRT improves LV function, creates a more efficient biventricular contraction, and reduces heart failure symptoms (Linde et al. 2002; Auricchio et al. 2002), hospitalizations, and mortality (Abraham et al. 2002; Cleland et al. 2005). Current guidelines support CRT with a class I recommendation for patients with systolic heart failure with LV ejection fraction (LVEF) ≤ 35% and prolonged ventricular depolarization on a 12‐lead electrocardiogram (ECG), that is, a combination of left bundle branch block (LBBB) and a long QRS duration (> 150 ms) (Brignole et al. 2013; Sipahi et al. 2011). By selecting CRT candidates with current guideline parameters, however, a significant portion of patients (30%–50%) do not derive the anticipated CRT response of symptomatic and echocardiographic improvement, that is, a reduction in LV end‐systolic volume (LVESV) or an improvement in LVEF (Abraham et al. 2002; Auricchio and Prinzen 2011; Bristow et al. 2004).

As current guideline parameters have limitations, additional ways of selecting suitable CRT candidates and estimating their prognosis with CRT are needed. Current parameters use a long QRS duration as an indication of prolonged LV depolarization (Brignole et al. 2013). However, a long QRS duration represents a long global myocardial activation time and measures both right and left ventricular conduction, rather than the long left ventricular activation time (LVAT) that CRT primarily aims to influence. An ECG measurement like LVAT may overcome this limitation of QRS duration, as it may more accurately reflect LV delay. Consistent with this hypothesis, previous research has found that a longer LVAT predicts reverse remodeling in patients with LBBB (Sweeney et al. 2010; Del‐Carpio Munoz et al. 2013). In addition, a retrospective study of 219 patients investigated the association between LVAT and clinical outcome and showed promising results (Eitel et al. 2012). The added value of LVAT measurements is currently unclear in patients with non‐LBBB ECG morphology. As LVAT is a readily available measurement from a standard 12‐lead surface ECG, it could be an easily accessible predictor of clinical outcome when assessing potential CRT candidates.

This study sought to evaluate the predictive value of pre‐ and post‐implant LVAT and changes in LVAT on long‐term clinical outcome in a population of patients treated with CRT.

## 2 Methods

### 2.1 Study Population and Baseline Data

The retrospective cohort consisted of consecutive patients undergoing CRT implantation at a tertiary care center at Skåne University Hospital in Sweden (CRT‐P or CRT‐D) from January 2015 through September 2020. The Swedish Ethical Review Authority approved this study, and the requirement for written informed consent was waived because the study used a registry‐based design.

Patients that fulfilled guideline criteria for CRT and had sufficient digital pre‐CRT ECG data were included. A narrow pre‐CRT QRS complex (< 120 ms), pacing prior to CRT implantation, failure to establish CRT, early device explantation (within 30 days), or follow‐up elsewhere (outside of the Southern Region of Sweden) excluded patients from further analysis.

Baseline data were retrieved from medical records, and baseline evaluation was constituted of anamnesis, physical examination, and a standard clinical evaluation entailing digital 12‐lead ECG, echocardiography, and blood tests measuring hemoglobin, N‐terminal pro–B‐type natriuretic peptide (NT‐proBNP), creatinine, and estimated glomerular filtration rate (eGFR). Heart failure etiology was considered ischemic in patients with a history of myocardial infarction, percutaneous coronary intervention (PCI), or coronary artery bypass grafting (CABG).

Throughout the manuscript, the terms “post‐implant” and “post‐CRT” denote the period following CRT implantation, that is, during CRT pacing.

### 2.2 Primary Endpoint Data

The primary combined endpoint for the study was heart failure hospitalization or death of cardiovascular or non‐cardiovascular causes. Endpoint data were obtained from the Swedish National Board of Health and Welfare, and the vital status of patients was verified as of January 2022.

### 2.3 Electrocardiographic Data

Standard 12‐lead ECG data were digitally collected pre‐ and post‐CRT implantation via the hospital's GE MUSE or EC View databases. ECGs were evaluated on morphology, QRS duration, and LVAT parameters. An experienced electrophysiologist (R.B.) used standard definitions of left bundle branch block (LBBB), intraventricular conduction delay (IVCD), and right bundle branch block (RBBB) when assessing and dividing all included ECGs into categories of LBBB, IVCD, or RBBB (Surawicz et al. 2009). This categorization was also validated by two experienced electrophysiologists (P.G. P.), and the interindividual correlation coefficient for identification of LBBB was 0.95 (*p* < 0.001).

### 2.4 Left Ventricular Activation Time (LVAT)

**FIGURE 1:**
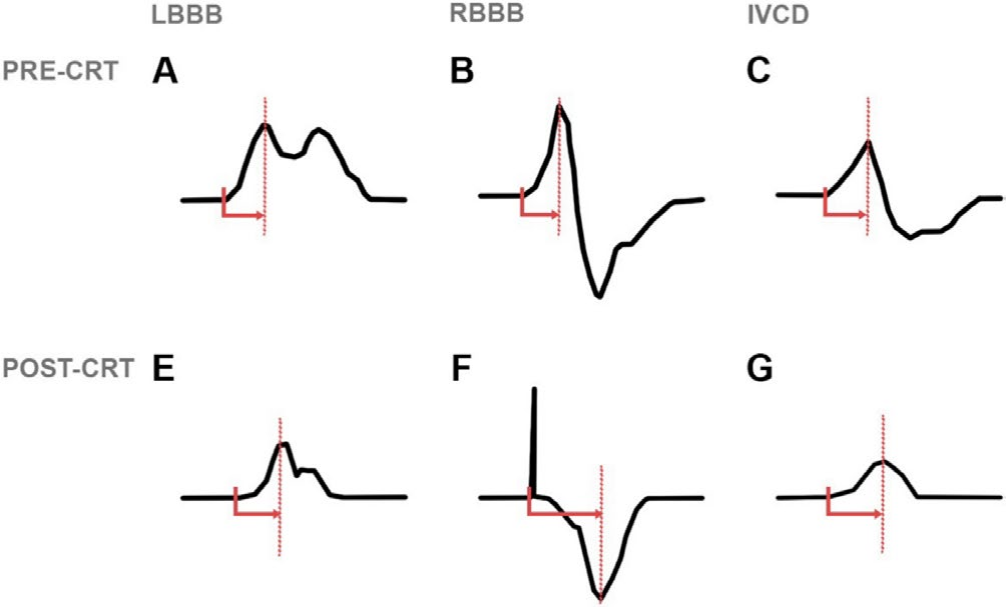
Examples of LVAT measurements in the different QRS morphologies. LVAT is measured as the time of QRS onset to maximum deflection in lead V6.

LVAT was manually calculated from digital pre‐ and post‐implant ECGs as the time interval between QRS onset and the maximal deflection toward the baseline in lead V6, as illustrated by Figure 1. This method was intended to provide a noninvasive approximation of the time required for depolarization to reach the lateral left ventricular free wall; hence, the choice of measuring LVAT in the laterally positioned V6 lead. This pragmatic method provides a simple and potentially clinically applicable estimate of LV activation time.

In cases where lead V6 exhibited multiple peaks, the calculation of LVAT was based on the largest peak. However, in instances where the maximum deflection in lead V6 significantly deviated from adjacent leads (V4 and V5), the calculation of LVAT in lead V6 was determined through guidance from adjacent leads. Assessment of intra‐ and interobserver variability in the calculation of LVAT was conducted in 40 randomly chosen patients; the intraindividual coefficient was 0.97 (*p* < 0.001), and the interindividual coefficient was 0.96 (*p* < 0.001).

### 2.5 Echocardiographic Data

All echocardiographic studies were obtained between 2015 and 2020 with an iE33 platform with an S5‐1 transducer (Philips Healthcare, Eindhoven, NL), and images were digitally stored into an echocardiographic database (Philips IntelliSpace Cardiovascular, Philips Healthcare, Eindhoven, NL). Echocardiographic analyzes were supported with IntelliSpace Cardiovascular (ISCV) and Xcelera software. For the present study, LVEF measurements were collected from two different types of digitally calculated EF: primarily MOD‐BP, and when MOD‐BP was not available, MOD‐SP4.

### 2.6 Statistical Analysis

Statistical analysis was conducted using SPSS Statistics version 26.0 (SPSS Inc., Chicago, IL, USA). A two‐sided *p*‐value < 0.05 was considered statistically significant. Normality was determined by visual inspection of histogram bars and by using the Kolmogorov–Smirnov test if needed. Normally distributed and continuous variables are presented with mean values and standard deviations. Non‐normally distributed data are presented with median values and interquartile ranges [Q1–Q3]. Categorical data are presented with numbers and percentages. Between‐group differences were assessed using the Chi‐square test, Kruskal‐Wallis test, or ANOVA as appropriate.

Kaplan–Meier analysis was used to construct survival curves for the risk of heart failure hospitalization or all‐cause mortality, and differences between the curves in the Kaplan–Meier graphs were tested for significance with the log‐rank test.

Uni‐ and multivariable Cox regression models were used to estimate hazard ratios for the risk of reaching the primary combined endpoint or for each endpoint analyzed separately (heart failure hospitalization or all‐cause mortality). Baseline variables included in the multivariable model were parameters known to be associated with CRT outcome—age, sex, CRT‐P or CRT‐D, secondary implantable cardioverter‐defibrillator (ICD) indication, ischemic heart failure etiology, New York Heart Association (NYHA) functional class, LVEF, diabetes, atrial fibrillation, NT‐proBNP, and eGFR. This predefined set of clinically relevant covariables was entered simultaneously into the multivariable Cox regression model. Multivariable hazard ratios and *p*‐values therefore represent the association of each variable with the outcome after adjustment for the other variables included in the model.

## 3 Results

### 3.1 Study Population and Baseline Characteristics

**FIGURE 2:**
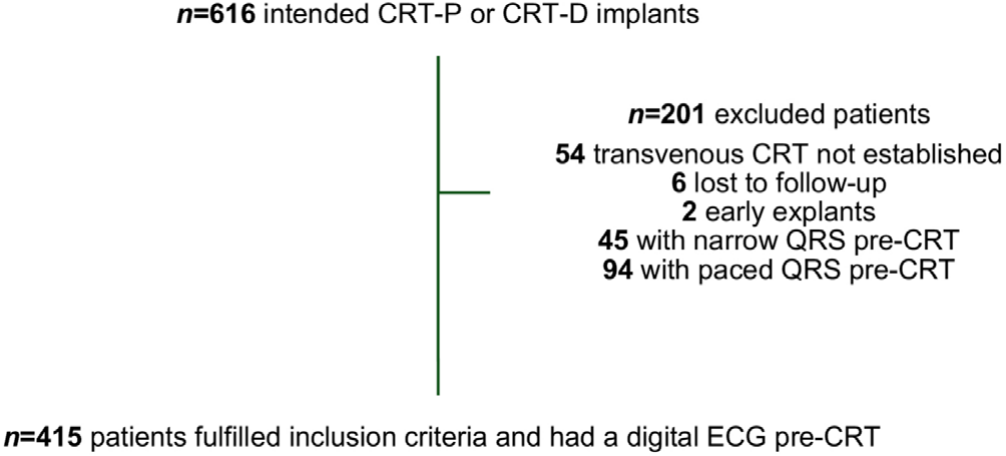
Flowchart of patient selection.

Six hundred and sixteen CRT implantations were identified between January 2015 and September 2020, of which 415 patients met inclusion criteria and had sufficient ECG data (Figure 2). Baseline demographic characteristics correlated well to typical CRT populations, with a median age of 72.8 years [IQR, 65.1–78.7] and a predominantly male cohort (77.3%) (Table 1). At baseline, LBBB morphology was present in 291 (70.1%), unspecific IVCD in 98 (23.6%), and RBBB (with or without bifascicular block) in 26 (6.3%) patients. Median LVEF was 27.5% [22–30] prior to CRT. There were no statistically significant differences in pre‐LVEF between patients with LBBB, IVCD, or RBBB (*p* = 0.42, see Table 2). Following CRT, the median LVEF was 32.5% [27.5–40.3], and the median change was an increase of 6.5% [0.0–14.1] in the entire cohort. Patients had high rates of biventricular pacing (median of 98% [94–99]) when assessed 6 months after implantation. Patients with chronic AF had similarly high rates of biventricular pacing with a median of 94% [84–98].

**TABLE 1:** Baseline characteristics.

Baseline characteristics, mean (SD) or median [IQR]	All patients	LBBB	IVCD	RBBB	*p* ^a^
	*n* = 415	*n* = 291	*n* = 98	*n* = 26	
Age at implantation, years, [IQR]	72.8 [65.1–78.7]	72.8 [64.8–78]	72.4 [65.1–77.5]	78 [69.3–83.4]	0.1^b^
Female sex, *n*, (%)	94 (22.7)	73 (25)	18 (18.4)	3 (11.5)	0.15^c^
CRT‐P or CRT‐D (primary or secondary), *n*, (%)					0.022^c^
CRT‐P	138 (33.3)	99 (34)	26 (26.5)	13 (50)	
CRT‐D (primary prophylactic)	244 (58.8)	175 (60.1)	58 (59.1)	11 (42.3)	
CRT‐D (secondary prophylactic)	33 (8)	17 (5.8)	14 (14.2)	2 (7.7)	
LVEF, % [IQR]	27.5 [22–30]	27.5 [22.5–30]	27.1 [20–30]	27.8 [22.5–35]	0.42^b^
NYHA class, *n*, (%)					0.94^c^
II	149 (38.9)	104 (35.7)	38 (38.8)	7 (26.9)	
III	197 (51.4)	139 (47.8)	45 (49.5)	13 (50)	
IV	27 (7.0)	19 (6.5)	6 (6.1)	2 (7.7)	
Ischemic etiology, *n*, (%)	179 (43.1)	117 (40.2)	53 (53.1)	10 (38.5)	0.075^c^
Hypertension, *n*, (%)	281 (67.7)	199 (68.4)	69 (23.7)	13 (50)	0.11^c^
Diabetes, *n*, (%)	137 (33.0)	100 (34.4)	28 (28.6)	9 (34.6)	0.59^c^
Previous PCI, *n*, (%)	135 (32.7)	88 (46.1)	37 (37.8)	10 (38.4)	0.3^c^
Previous CABG, *n*, (%)	78 (18.9)	50 (17.2)	24 (24.5)	4 (15.4)	0.24^c^
Atrial fibrillation, *n*, (%)					0.011^c^
Chronic	98 (23.7)	56 (19.2)	33 (33.6)	9 (34.6)	
Paroxysmal	92 (22.2)	62 (21.3)	23 (23.4)	7 (26.9)	
No	224 (54.1)	172 (59.1)	42 (42.9)	10 (38.5)	
Hemoglobin, mg/L (SD)	133.6 (16.5)	133.4 (16.3)	133.9 (15.8)	131.5 (19.1)	0.99^d^
NT‐proBNP, ng/L [IQR]	1711 [713–3937]	1367 [617–3282]	2462 [771–5888]	1985 [1028–3910]	0.03^b^
Creatinine, g/dL [IQR]	103 [83–133]	100 [81–136]	101 [88–130]	115 [90–134]	0.75^b^
eGFR, mL/min/m^2^ (SD)	53.9 (20.0)	55.5 (19.6)	55.8 (20.3)	53.6 (22.7)	0.95^d^
Beta blocker use, *n*, (%)	347 (83.6)	248 (85.2)	82 (83.6)	18 (69.2)	0.032^c^
ACE‐inhibitor or Angiotensin‐receptor blocker use, *n*, (%)	327 (78.8)	235 (80.8)	74 (75.5)	19 (73.1)	0.26^c^
Sacubitril/Valsartan use, *n*, (%)	53 (12.8)	32 (11)	17 (17.3)	4 (15.4)	0.24^c^
Aldosterone antagonist use, *n*, (%)	224 (54)	160 (54.9)	51 (52)	13 (50)	0.81^c^
Loop diuretic use, *n*, (%)	257 (64.3)	187 (64.3)	62 (63.2)	18 (69.2)	0.85^c^
Class I or III antiarrhythmic use, *n*, (%)	18 (4.3)	6 (2)	6 (6.1)	6 (23.1)	< 0.001^c^
Digoxin use, *n*, (%)	35 (8.4)	19 (6.5)	14 (14.2)	2 (7.7)	0.057^c^
Anticoagulant use, *n*, (%)	196 (47.2)	119 (41)	60 (61.2)	17 (65.4)	< 0.001^c^
Previous pacemaker or ICD, *n*, (%)	47 (11.3)	23 (7.9)	16 (16.3)	8 (30.7)	< 0.001^c^
Time to follow‐up, years, [IQR]	2.8 [1.7–4.2]	3.0 [2–4.4]	2.5 [0.9–3.6]	2.7 [1.3–4.7]	< 0.001^b^

Abbreviations: ACE indicates angiotensin‐converting enzyme; CABG, coronary artery bypass grafting; CRT, cardiac resynchronization therapy; ICD, implantable cardioverter defibrillator; LVEF, left ventricular ejection fraction; NT‐proBNP, N‐terminal pro–B‐type natriuretic peptide; NYHA, New York Heart Association; PCI, percutaneous coronary intervention.

^a^ Comparisons were made between LBBB, IVCD, and RBBB.

^b^ Kruskal–Wallis test.

^c^ Chi‐square test.

^d^ ANOVA.

**TABLE 2:** Electro‐ and echocardiographic characteristics.

Patient characteristics, mean (SD) or median [IQR]	All patients	LBBB	IVCD	RBBB	*p* ^a^
	*n* = 415	*n* = 291	*n* = 98	*n* = 26	
ECG characteristics					
Pre‐QRSd, ms (SD)	163.9 (19.7)	165.4 (17.7)	157.3 (22.2)	170.9 (25.8)	< 0.001^b^
Post‐QRSd, ms (SD)	157.1 (24.7)	156.3 (24.7)	157.9 (24.9)	163.1 (24.7)	0.38^b^
ΔQRSd, ms (SD)	−6.8 (25.8)	−9.3 (24.9)	0.3 (26.3)	−7.8 (28.8)	0.01^b^
Pre‐LVAT, ms [IQR]	78 [66–98]	80 [68–98]	75 [60–98]	77 [62–109]	0.12^c^
Post‐LVAT, ms [IQR]	88 [74–106]	88 [74–106]	90 [74.5–108]	89 [73.5–100]	0.99^c^
ΔLVAT, ms [IQR]	8 [−12–30]	8 [−14–28]	13 [−5.5–32]	9 [−23–34]	0.5^c^
Echo characteristics					
Pre‐LVEF, % [IQR]	27.5 [22.4–30]	27.5 [22.5–30]	27.1 [20–30]	27.8 [22.5–35]	0.42^c^
Post‐LVEF, % [IQR]	32.5 [27.5–40.3]	35 [28.6–42.5]	30 [24.6–35]	31.9 [22.5–37.5]	< 0.001^c^
ΔLVEF, % [IQR]	6.5 [0.0–14.1]	8.8 [1.15–17.3]	2.5 [−1–9.5]	1 [−0.6–7.5]	< 0.001^c^

Abbreviations: ECG indicates electrocardiogram; IVCD, intraventricular conduction delay; LBBB, left bundle branch block; LVAT, left ventricular activation time; QRSd, QRS duration; RBBB, right bundle branch block.

^a^ Comparisons were made between LBBB, IVCD, and RBBB.

^b^ ANOVA.

^c^ Kruskal–Wallis test.

Prior to CRT implantation, the median LVAT was 78 ms [66–98]. During CRT, the median LVAT was 88 ms [74–106] (Table 2). There was a post‐CRT increase of LVAT with a median of 8 ms [−12–30] (*p* < 0.001). Mean QRS duration decreased significantly (*p* < 0.001) from 163.9 (19.7) ms pre‐implant to 157.1 (24.7) ms post‐implant, with a mean decrease of 6.8 (25.8) ms (Table 2).

There was a relatively high correlation between pre‐implant LVAT and pre‐implant QRS duration of 0.6 (*p* < 0.001). Owing to this, pre‐implant QRS duration was not entered as a covariable in the multivariable Cox regression model of pre‐implant LVAT and vice versa.

### 3.2 Clinical Outcome

Over a median follow‐up time of 2.8 years [1.7–4.2], 171 patients reached the primary combined endpoint of heart failure hospitalization (*n* = 122) and/or all‐cause mortality (*n* = 113), of which most deaths were cardiovascular (*n* = 68).

In the following presented data, analysis of the entire cohort was performed with the exclusion of RBBB patients (*n =* 26). Patients with RBBB were excluded in these analyses since their inclusion was expected to cloud the analysis of the total cohort. This decision was based on findings from Kaplan–Meier analysis, which demonstrated that the analysis of pre‐implant LVAT in patients with RBBB exhibited a divergent trend in comparison to the rest of the cohort (patients with LBBB and IVCD), see Figure S3.

### 3.3 LVAT

#### 3.3.1 Pre‐Implant

Cox regression analysis was used to assess pre‐ and post‐CRT LVAT and change in LVAT for the uni‐ and multivariable association with the primary endpoint (Table 3). In univariable analysis of all patients with LBBB and IVCD, patients with a longer pre‐implant LVAT had a reduced risk of reaching the combined endpoint of heart failure hospitalization and all‐cause mortality (HR, 0.92 [0.86–0.99]; *p* = 0.026). This correlation was of borderline significance in subsequent multivariable analysis (HR, 0.93 [0.85–1.01]; *p* = 0.086). Table 4 presents a full multivariable Cox regression model adjusted for clinically relevant covariables, that is, age, sex, CRT‐P or CRT‐D, secondary ICD indication, ischemic etiology, NYHA class, LVEF, diabetes, atrial fibrillation, NT‐proBNP, and eGFR. In a sub‐analysis of all patients with LBBB and IVCD with a positive deflection for LVAT (Table S4), pre‐implant LVAT was univariably associated with the primary endpoint (HR, 0.89 [0.81–0.96]; *p* = 0.005). Additional Cox regression analyzes using median‐dichotomized pre‐implant LVAT are presented in Table 3. Pre‐implant LVAT showed a significant association with the endpoint in univariable analysis, but not after multivariable adjustment.

**TABLE 3:** Cox regression analysis for prediction of the primary endpoint (heart failure hospitalization or all‐cause mortality) in all patients with LBBB and IVCD (*n* = 389).

Parameter	Univariable			Multivariable^a^		
	HR	95% CI	*p*	HR	95% CI	*p*
QRS duration						
Pre‐QRSd (per 10 ms)	0.87	0.80–0.95	0.001	0.86	0.78–0.95	0.003
Pre‐QRSd ≥ median (158 ms)	0.69	0.50–0.93	0.017	0.63	0.43–0.93	0.02
Post‐QRSd (per 10 ms)	1.1	1.04–1.17	0.001	1.04	0.96–1.13	0.31
ΔQRSd (per 10 ms decrease)	0.84	0.79–0.89	< 0.001	0.89	0.82–0.95	0.001
LVAT						
Pre‐LVAT (per 10 ms)	0.92	0.86–0.99	0.026	0.93	0.85–1.01	0.086
Pre‐LVAT ≥ median (80 ms)	0.69	0.51–0.94	0.019	0.76	0.52–1.12	0.17
Post‐LVAT (per 10 ms)	1.01	0.95–1.08	0.75	1.03	0.95–1.11	0.49
ΔLVAT (per 10 ms increase)	1.06	1.001–1.11	0.046	1.06	0.99–1.13	0.067

Abbreviations: CRT indicates cardiac resynchronization therapy; LVAT, left ventricular activation time.

^a^ Baseline variables included in the multivariable model were age, sex, CRT‐P or CRT‐D, secondary ICD indication, ischemic etiology, NYHA class, LVEF, diabetes, atrial fibrillation, NT‐proBNP, and eGFR.

**TABLE 4:** Full multivariable model for prediction of the primary endpoint (heart failure hospitalization or all‐cause mortality) in all patients with LBBB and IVCD (*n* = 389).

Parameter	Multivariable^a^		
	HR	95% CI	*p*
Pre‐LVAT (per 10 ms increase)	0.93	0.85–1.01	0.086
Age (per 1 year increase)	0.97	0.95–0.99	0.033
Sex (male reference)	0.54	0.32–0.91	0.02
CRT‐P or CRT‐D (CRT‐P reference)	0.99	0.60–1.63	0.97
Primary or secondary CRT‐D indication (primary prophylactic reference)	1.03	0.52–2.04	0.93
Ischemic etiology	1.52	1.01–2.28	0.04
NYHA class (NYHA class I reference)			
NYHA class II	0.43	0.12–1.48	0.18
NYHA class III	0.82	0.24–2.86	0.75
NYHA class IV	0.82	0.21–3.3	0.79
LVEF baseline (per absolute % increase)	0.98	0.95–1.01	0.14
Diabetes	0.86	0.57–1.31	0.48
Atrial fibrillation			
Paroxysmal	1.26	0.79–1.97	0.34
Chronic	1.35	0.82–2.22	0.24
NT‐proBNP (per 1000 ng/L increase)	1.00	1.00–1.00	0.003
eGFR (per mL/min/m^2^ increase)	0.99	0.98–1.00	0.056

Abbreviations: CRT indicates cardiac resynchronization therapy; ECG, electrocardiogram; LVEF, left ventricular ejection fraction; NT‐proBNP, N‐terminal pro–B‐type natriuretic peptide; NYHA, New York Heart Association.

^a^ Baseline variables included in the multivariable model were age, sex, CRT‐P or CRT‐D, secondary ICD indication, ischemic etiology, NYHA class, LVEF, diabetes, atrial fibrillation, NT‐proBNP, and eGFR.

**FIGURE 3:**
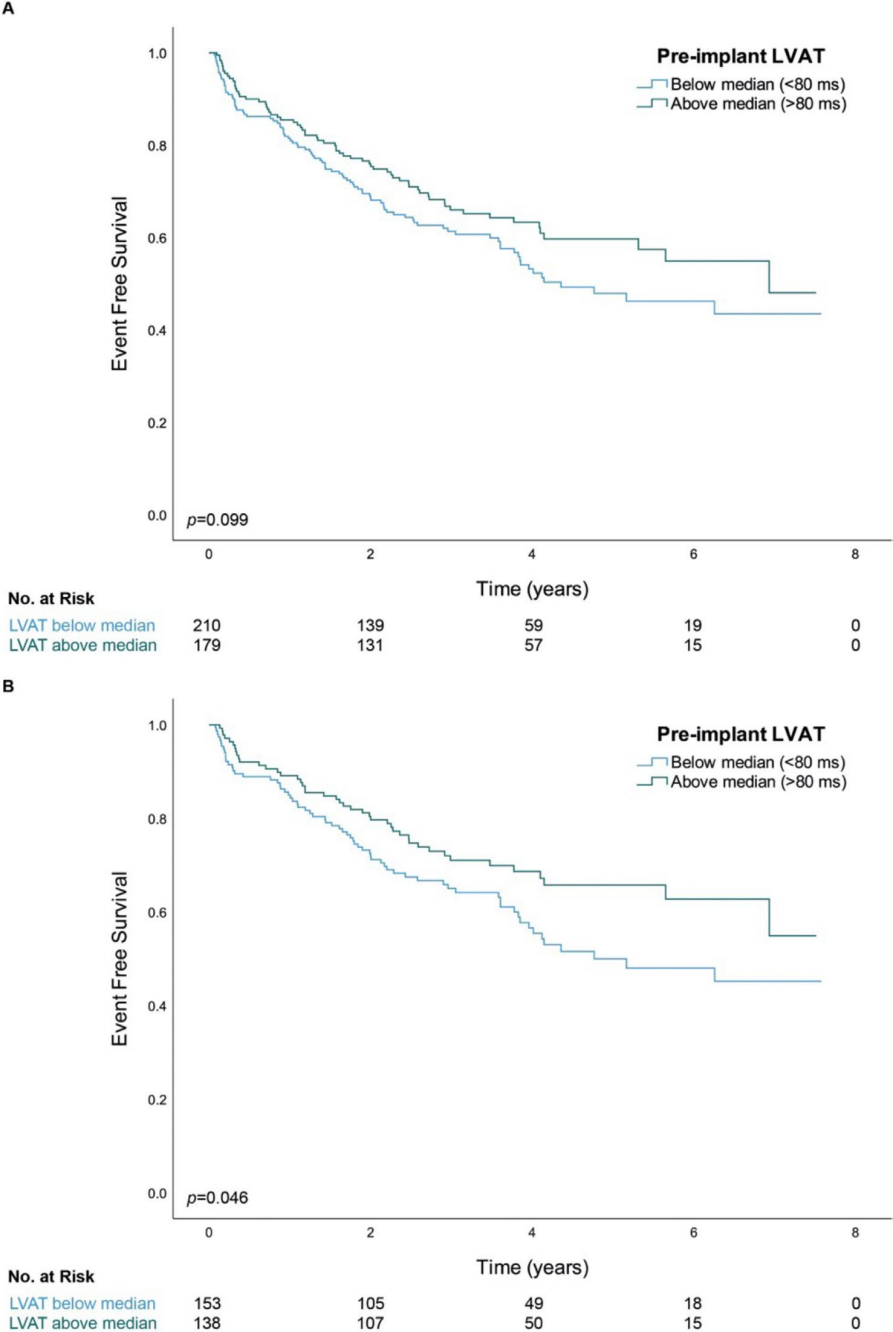
Kaplan–Meier curves of survival free of heart failure hospitalization stratified by the median pre‐implant LVAT in (A) all patients with LBBB and IVCD (*n* = 389) and (B) all patients with LBBB (*n* = 291).

In Kaplan–Meier analysis of all patients with LBBB and IVCD, dichotomized based on the pre‐implant median LVAT (80 ms in the LBBB and IVCD group), patients with a longer pre‐implant LVAT had a borderline significant risk reduction of heart failure hospitalization and all‐cause mortality (Figure 3A, log‐rank *p* = 0.099). In LBBB patients, a longer pre‐implant LVAT was significantly associated with a reduced risk of reaching the primary endpoint (Figure 3B, log‐rank *p* = 0.046). In a sub‐analysis of all patients with LBBB and IVCD and a positive deflection for LVAT (Figure S1), there was a significantly lower risk of reaching the endpoint (*p* = 0.01).

#### 3.3.2 Post‐Implant

In analysis of all patients with LBBB and IVCD, a longer post‐implant LVAT was not associated with an increased risk of heart failure hospitalization or all‐cause mortality (HR, 1.01 [0.95–1.08]; *p* = 0.75). A post‐implant increase of LVAT was associated with an increased risk of reaching the primary endpoint (univariable HR, 1.06 [1.001–1.11]; *p* = 0.046). This finding was of borderline significance in subsequent multivariable analysis (HR, 1.06 [0.99–1.13]; *p* = 0.067).

### 3.4 LVAT in Different ECG Morphologies

Differences in the predictive value of LVAT were explored in the different ECG morphology groups by analyzing them separately for the association with the primary endpoint.

#### 3.4.1 LBBB Morphology

In univariable Cox regression analysis of patients with native LBBB (Table S5), there was a significant association between pre‐implant LVAT and the primary endpoint of heart failure hospitalization and all‐cause mortality (HR, 0.9 [0.82–0.99]; *p* = 0.03). In multivariable analysis, however, the association between a longer pre‐implant LVAT and the primary endpoint was not significant (HR, 0.92 [0.82–1.04]; *p* = 0.17). A longer post‐implant LVAT (HR, 0.99 [0.91–1.07]; *p* = 0.79) or a larger increase of LVAT following CRT (HR, 1.05 [0.98–1.12]; *p* = 0.16) did not correlate with the primary endpoint in patients with LBBB.

#### 3.4.2 Non‐LBBB Morphology

In Kaplan–Meier analysis of patients with IVCD (Figure S2) or RBBB (Figure S3), there were no associations with the primary endpoint (log‐rank *p* = 0.52 and log‐rank *p* = 0.56, respectively). In uni‐ and multivariable Cox regression analyses of patients with IVCD, neither a longer pre‐implant LVAT (univariable HR, 0.97 [0.88–1.08]; *p* = 0.63) nor a longer post‐implant LVAT (univariable HR, 1.07 [0.96–1.21]; *p* = 0.28) correlated with the primary endpoint (Table S6).

In univariable analysis, there was no significant association between pre‐implant LVAT and the primary endpoint in patients with RBBB morphology (HR, 1.13 [0.95–1.35]; *p* = 0.18), see Table S7. There was an association between a longer post‐implant LVAT (HR, 0.55 [0.33–0.91]; *p* = 0.02) and a larger increase of LVAT (HR, 0.82 [0.69–0.97]; *p* = 0.02) and a decreased risk of reaching the primary endpoint. The subset of patients with RBBB (*n* = 26) was too small for further analysis in the multivariable model.

## 4 Discussion

### 4.1 Main Findings

The present study investigated the predictive value of LVAT on long‐term clinical outcome following CRT. The main findings were that a longer baseline LVAT was associated with a lower risk of heart failure hospitalization and all‐cause mortality in analysis of all patients with LBBB and IVCD and in separate analysis of patients with LBBB. In these groups, patients with the longest baseline LVAT had the lowest risk of reaching the endpoint, while the reverse, that is, the highest risk, was observed in patients with the shortest pre‐implant LVAT.

### 4.2 LVAT in Relation to Clinical Endpoints

The use of delayed LV activation time to predict CRT response has been studied previously (Sweeney et al. 2010). In a prospective study of 202 patients, Sweeney et al. used an intricate analysis of the surface 12‐lead ECG to estimate the baseline LV conduction delay and the LV activation sequence prior to and following CRT implantation. The main finding regarding LVAT was that a larger maximum LVAT was associated with a higher likelihood of reverse remodeling following CRT.

Del‐Carpio et al. (Del‐Carpio Munoz et al. 2013) estimated LVAT using the R‐peak time in leads I, aVL, V1, V2, V5, and V6 in 135 patients selected for CRT implantation with current guideline criteria. CRT response was defined as a decrease in LVESV > 15% at 6 months. It was found that a longer LVAT predicted response to CRT and was a better predictor of echocardiographic response than QRS duration in patients with LBBB and IVCD morphology.

In addition to the studies on echocardiographic response, a smaller retrospective study on the predictive value of LVAT on long‐term clinical outcome was performed by Eitel et al. on 219 patients (Eitel et al. 2012). The study included chronically right ventricular (RV) paced and non‐paced patients. LVAT was measured as QRS duration subtracted with the time from QRS onset to the first notch in the QRS. It was found that a longer LVAT (≥ 125 ms) was associated with a markedly lower risk of reaching the combined primary endpoint of death or cardiac transplantation. In patients with non‐LBBB morphology, however, there was no significant association with the endpoint.

In a study by Dural et al. (2021) on a non‐LBBB population of 790 patients, LVAT was measured as the time between the first notch in any of the leads V1, V2, I, avL, V5, or V6 and the end of the QRS complex. Patients were stratified into groups of LVAT above or below 125 ms. In Cox regression analysis, there was no significant association between LVAT and the primary nor secondary endpoint in this cohort of patients with non‐LBBB morphology. The primary endpoint was a combination of left ventricular assist device (LVAD) implantation, cardiac transplantation, and all‐cause mortality, and the secondary endpoint was echocardiographic reduction of LVESV.

Building on the studies presented above, our data provide some support for the hypothesis that estimating LVAT on a standard 12‐lead ECG permits a noninvasive method of estimating long‐term clinical outcome following CRT in patients with LBBB and IVCD. Our proposed method of estimating LVAT on lead V6 has the advantage of being simpler to perform, thereby increasing its potential clinical applicability.

### 4.3 LVAT in Comparison to Current ECG Parameters: ECG Morphology and QRS Duration

Current guidelines utilize ECG morphology and pre‐implant QRS duration to measure the biventricular dyssynchrony that CRT aims to correct. Many studies suggest that QRS duration is a clinically effective marker (Cleland et al. 2005; Bristow et al. 2004; Gold et al. 2012). Nevertheless, some studies have shown limitations in predicting response with QRS duration (Sipahi et al. 2011; Stavrakis et al. 2012). It can be speculated that delayed LVAT displays left intraventricular electrical dyssynchrony better than QRS duration and, therefore, might be of additional value in selecting CRT candidates. The results of the present study suggest that LVAT is a univariable predictor of CRT response in patients with LBBB and non‐LBBB morphology. In multivariable analysis, however, it did not perform better than QRS duration.

### 4.4 Clinical Implications

Accurate predictors of prognosis are valuable in the clinical setting; better patient selection criteria may increase the proportion of responders as well as reduce the number of non‐responders. In the present study, a prolonged LVAT was a predictor of a beneficial CRT outcome. Ideally, this data could be presented as an optional automatic calculation for heart failure patients by the electronic medical record system. It should be noted, however, that it did not perform better than QRS duration, which is the current patient selection parameter for CRT.

Current medical record systems frequently lack the necessary software and convenient access to digital ECG data. As such, integrating alternative ECG measurements in patient selection for CRT poses a challenge. Should medical record systems enable accessing this information in a straightforward way, however, LVAT and other alternative ECG parameters could be more widely used in addition to current guidelines. In this context, randomized trials that further investigate the additional prognostic information from LVAT measurements compared to conventional selection parameters are warranted.

### 4.5 Limitations

The present study has inherent limitations owing to the retrospective study design (selection, referral, and attrition biases); prospective randomized controlled trials are required to determine the absolute predictive value of LVAT parameters on clinical outcome following CRT. The multivariable statistical models included previously identified variables that influence CRT outcome. However, there is a possibility of residual bias that was not adequately accounted for in the statistical analyses.

LVAT determination becomes less straightforward during biventricular pacing because of pacing induced electrophysiological changes; the inspection of all post‐implant LVAT assessments indicated a larger heterogeneity in LVAT during CRT compared to pre‐CRT. In addition, varying degrees of pace latency may have further complicated the assessment of post‐implant LVAT.

The cohort size of 415 patients was relatively small. Hence, conclusions about the predictive value of LVAT in ECG morphology groups with few patients (e.g., RBBB) should be drawn with caution. All CRT implanters were experienced and aimed for a posterolateral/lateral‐, mid‐, or basal LV lead position. However, detailed information on LV lead position was not available. This may have affected the results as LV lead position may have a different impact on LVAT in different ECG morphologies.

## 5 Conclusion

In patients with heart failure treated with CRT, a longer baseline LVAT was associated with a reduced risk of heart failure hospitalization and mortality. As a straightforward ECG‐derived parameter, LVAT offers a noninvasive means of assessment and may better reflect the underlying substrate amenable to CRT in some patients. It should be noted, however, that in this cohort LVAT did not outperform QRS duration, which is the current patient selection parameter for CRT. In this context, prospective trials would be useful to further explore the potential role of pre‐implant LVAT in patient selection for CRT.

## Author Contributions

**Sofia Marinko:** conceptualization, data collection, data curation, formal analysis, and writing the original draft and reviewing the manuscript. **Rasmus Borgquist:** conceptualization, data collection, formal analysis, and review of manuscript. **Pyotr G. Platonov:** data collection, formal analysis, and review of manuscript.

## Funding

This work was supported by the Hjärt‐Lungfonden, the Anna‐Lisa and Sven Eric Lundgren Foundation, and the ALF grants (within the Swedish health care system).

## Ethics Statement

The Swedish Ethical Review Authority provided ethical approval for this study. The requirement of written informed consent was waived by the authority. Approval number: 2020–05843.

## Conflicts of Interest

R.B. is an employee at the Novo Nordisk Foundation and has received speaker fees from BIOTRONIK and Medtronic; there are no conflicts of interest relevant to the presented manuscript. P.G.P. is a member of Advisory Board at Tenaya Therapeutics and received speaker fees from Pfizer and Bristol Myers Squibb; there are no conflicts of interest relevant to the presented manuscript. S.M. declares no conflicts of interest.

## Supporting information


**Figure S1:** Kaplan‐Meier curve of survival free of heart failure hospitalization stratified by median pre‐implant LVAT in all patients with LBBB and IVCD with a positive deflection for LVAT (*n* = 336).
**Figure S2:** Kaplan‐Meier curve of survival free of heart failure hospitalization stratified by median pre‐implant LVAT in patients with IVCD (*n* = 98).
**Figure S3:** Kaplan‐Meier curve of survival free of heart failure hospitalization stratified by median pre‐implant LVAT in patients with RBBB (*n* = 26).
**Table S4:** Cox regression analysis for prediction of the primary endpoint (heart failure hospitalization or death) in all patients with LBBB and IVCD with a positive deflection for LVAT (*n* = 336).
**Table S5:** Cox regression analysis for prediction of the primary endpoint (heart failure hospitalization or death) in patients with LBBB (*n* = 291).
**Table S6:** Cox regression analysis for prediction of the primary endpoint (heart failure hospitalization or death) in patients with IVCD (*n* = 98).
**Table S7:** Cox regression analysis for prediction of the primary endpoint (heart failure hospitalization or death) in patients with RBBB (*n* = 26).

## Data Availability

Data can be obtained by making a reasonable request to the corresponding author (S.M.). The data have not been made publicly available in order to protect the confidentiality and privacy of the individuals who took part in this study.
