# Supplementary material for: Longer Baseline Left Ventricular Activation Time Is Associated With Lower Mortality and Lower Risk of Heart Failure Hospitalization in Cardiac Resynchronization Therapy Recipients
Source: Ann Noninvasive Electrocardiol. 2026 Jun 10;31(4):e70210. doi: 10.1111/anec.70210 (PMC13253996; doi:10.1111/anec.70210)
Supplement: Supplementary file 1 — Figure S1: Kaplan‐Meier curve of survival free of heart failure hospitalization stratified by median pre‐implant LVAT in all patients with LBBB and IVCD with a positive deflection for LVAT (n = 336). Figure S2: Kaplan‐Meier curve of survival free of heart failure hospitalization stratified by median pre‐implant LVAT in patients with IVCD (n = 98). Figure S3: Kaplan‐Meier curve of survival free of heart failure hospitalization stratified by median pre‐implant LVAT in patients with RBBB (n = 26). Table S4: Cox regression analysis for prediction of the primary endpoint (heart failure hospitalization or death) in all patients with LBBB and IVCD with a positive deflection for LVAT (n = 336). Table S5: Cox regression analysis for prediction of the primary endpoint (heart failure hospitalization or death) in patients with LBBB (n = 291). Table S6: Cox regression analysis for prediction of the primary endpoint (heart failure hospitalization or death) in patients with IVCD (n = 98). Table S7: Cox regression analysis for prediction of the primary endpoint (heart failure hospitalization or death) in patients with RBBB (n = 26). [file ANEC-31-e70210-s001.docx]

**Supplementary Material**

**Contents:

Supplementary material for Figure 3:

S1.** Kaplan-Meier curve of survival free of heart failure hospitalization stratified by median pre-implant LVAT in all patients with LBBB and IVCD with a positive deflection for LVAT (*n*=336).

**S2.** Kaplan-Meier curve of survival free of heart failure hospitalization stratified by median pre-implant LVAT in patients with IVCD (*n*=98). **S3.** Kaplan-Meier curve of survival free of heart failure hospitalization stratified by median pre-implant LVAT in patients with RBBB (*n*=26).

**Supplementary material for Table 3:**

**S4.** Cox regression analysis for prediction of the primary endpoint (heart failure hospitalization or death) in all patients with LBBB and IVCD with a positive deflection for LVAT (*n*=336). **S5.** Cox regression analysis for prediction of the primary endpoint (heart failure hospitalization or death) in patients with LBBB (*n*=291). **S6.** Cox regression analysis for prediction of the primary endpoint (heart failure hospitalization or death) in patients with IVCD (*n*=98).

**S7.** Cox regression analysis for prediction of the primary endpoint (heart failure hospitalization or death) in patients with RBBB (*n*=26).

**Supplementary material for Figure 3:**

**S1.** Kaplan-Meier curve of survival free of heart failure hospitalization stratified by median pre-implant LVAT in all patients with LBBB and IVCD with a positive deflection for LVAT (*n*=336).

**S2.** Kaplan-Meier curve of survival free of heart failure hospitalization stratified by median pre-implant LVAT in patients with IVCD (*n*=98).

**S3.** Kaplan-Meier curve of survival free of heart failure hospitalization stratified by median pre-implant LVAT in patients with RBBB (*n*=26).

**Supplementary material for Table 3:**

**S4.** Cox regression analysis for prediction of the primary endpoint (heart failure hospitalization or death) in all patients with LBBB and IVCD with a positive deflection for LVAT (*n*=336).

|  | **Univariable** | | | **Multivariable*** | | |
| --- | --- | --- | --- | --- | --- | --- |
| **Parameter** | **HR** | **95% CI** | ***p*** | **HR** | **95% CI** | ***p*** |
| Pre-QRSd (per 10 ms) | 0.86 | 0.78–0.94 | 0.001 | 0.86 | 0.77–0.96 | 0.006 |
| Post-QRSd (per 10 ms) | 1.09 | 1.02–1.16 | 0.013 | 1.03 | 0.95–1.12 | 0.48 |
| ΔQRSd (per 10 ms decrease) | 0.85 | 0.80–0.91 | <.001 | 0.87 | 0.83–0.97 | 0.005 |
| Pre-LVAT (per 10 ms) | 0.89 | 0.81–0.96 | 0.005 | 0.92 | 0.83–1.01 | 0.079 |
| Post-LVAT (per 10 ms) | 1.004 | 0.93–1.08 | 0.92 | 1.04 | 0.96–1.13 | 0.35 |
| ΔLVAT (per 10 ms increase) | 1.07 | 1.01–1.14 | 0.03 | 1.08 | 1.004–1.15 | 0.04 |

CRT indicates cardiac resynchronization therapy; LVAT, left ventricular activation time; and QRSd, QRS duration.
*Baseline variables included in the multivariable model were age, gender, CRT-P or CRT-D, secondary ICD indication, ischemic etiology, NYHA class, LVEF, diabetes, atrial fibrillation, NT-proBNP, and eGFR.

**S5.** Cox regression analysis for prediction of the primary endpoint (heart failure hospitalization or death) in patients with LBBB (*n*=291).

|  | **Univariable** | | | **Multivariable*** | | |
| --- | --- | --- | --- | --- | --- | --- |
| **Parameter** | **HR** | **95% CI** | ***p*** | **HR** | **95% CI** | ***p*** |
| Pre-QRSd (per 10 ms) | 0.88 | 0.79–0.98 | 0.02 | 0.87 | 0.76–1.004 | 0.058 |
| Post-QRSd (per 10 ms) | 1.1 | 1.02–1.18 | 0.01 | 1.08 | 0.99–1.18 | 0.067 |
| ΔQRSd (per 10 ms decrease) | 0.85 | 0.79–0.92 | <.001 | 0.88 | 0.8–0.95 | 0.002 |
| Pre-LVAT (per 10 ms) | 0.9 | 0.82–0.99 | 0.03 | 0.92 | 0.82–1.04 | 0.17 |
| Post-LVAT (per 10 ms) | 0.99 | 0.91–1.07 | 0.79 | 1.055 | 0.96–1.1 | 0.26 |
| ΔLVAT (per 10 ms increase) | 1.05 | 0.98–1.12 | 0.16 | 1.07 | 0.99–1.15 | 0.07 |

CRT indicates cardiac resynchronization therapy; LVAT, left ventricular activation time; and QRSd, QRS duration.
*Baseline variables included in the multivariable model were age, gender, CRT-P or CRT-D, secondary ICD indication, ischemic etiology, NYHA class, LVEF, diabetes, atrial fibrillation, NT-proBNP, and eGFR.

**S6.** Cox regression analysis for prediction of the primary endpoint (heart failure hospitalization or death) in patients with IVCD (*n*=98).

|  | **Univariable** | | | **Multivariable*** | | |
| --- | --- | --- | --- | --- | --- | --- |
| **Parameter** | **HR** | **95% CI** | ***p*** | **HR** | **95% CI** | ***p*** |
| Pre-QRSd (per 10 ms) | 0.92 | 0.81–1.04 | 0.19 | 0.88 | 0.77–1.002 | 0.093 |
| Post-QRSd (per 10 ms) | 1.13 | 1.01–1.26 | 0.04 | 1.00 | 0.84–1.19 | 0.99 |
| ΔQRSd (per 10 ms decrease) | 0.85 | 0.76–0.95 | 0.003 | 0.88 | 0.76–1.03 | 0.11 |
| Pre-LVAT (per 10 ms) | 0.97 | 0.88–1.08 | 0.63 | 0.99 | 0.98–1.01 | 0.50 |
| Post-LVAT (per 10 ms) | 1.07 | 0.96–1.21 | 0.28 | 1.001 | 0.99–1.02 | 0.88 |
| ΔLVAT (per 10 ms increase) | 1.07 | 0.97–1.18 | 0.17 | 1.04 | 0.91–1.18 | 0.61 |

CRT indicates cardiac resynchronization therapy; LVAT, left ventricular activation time; and QRSd, QRS duration.
*Baseline variables included in the multivariable model were age, gender, CRT-P or CRT-D, secondary ICD indication, ischemic etiology, NYHA class, LVEF, diabetes, atrial fibrillation, NT-proBNP, and eGFR.

**S7.** Cox regression analysis for prediction of the primary endpoint (heart failure hospitalization or death) in patients with RBBB (*n*=26).

|  | **Univariable** | | | **Multivariable*** | | |
| --- | --- | --- | --- | --- | --- | --- |
| **Parameter** | **HR** | **95% CI** | ***p*** | **HR** | **95% CI** | ***p*** |
| Pre-QRSd (per 10 ms) | 1.27 | 0.97–1.67 | 0.09 | - | - | - |
| Post-QRSd (per 10 ms) | 0.99 | 0.74–1.33 | 0.97 | - | - | - |
| ΔQRSd (per 10 ms decrease) | 1.22 | 0.95–1.56 | 0.13 | - | - | - |
| Pre-LVAT (per 10 ms) | 1.13 | 0.95–1.35 | 0.18 | - | - | - |
| Post-LVAT (per 10 ms) | 0.55 | 0.33–0.91 | 0.02 | - | - | - |
| ΔLVAT (per 10 ms increase) | 0.82 | 0.69–0.97 | 0.02 | - | - | - |

CRT indicates cardiac resynchronization therapy; LVAT, left ventricular activation time; and QRSd, QRS duration.
*Baseline variables included in the multivariable model were age, gender, CRT-P or CRT-D, secondary ICD indication, ischemic etiology, NYHA class, LVEF, diabetes, atrial fibrillation, NT-proBNP, and eGFR.
